# Growing health: global linkages between patterns of food supply, sustainability, and vulnerability to climate change

**DOI:** 10.1016/S2542-5196(22)00223-6

**Published:** 2022-11-01

**Authors:** Rosemary Green, Pauline Scheelbeek, James Bentham, Soledad Cuevas, Pete Smith, Alan D Dangour

**Affiliations:** Department of Population Health; Centre on Climate Change and Planetary Health; Department of Population Health; Centre on Climate Change and Planetary Health; London School of Hygiene & Tropical Medicine, London, UK; School of Mathematics, Statistics and Actuarial Science, University of Kent, Canterbury, UK; Department of Population Health; Institute of Biological and Environmental Sciences, School of Biological Sciences, University of Aberdeen, Aberdeen, UK; Department of Population Health; Centre on Climate Change and Planetary Health

## Abstract

Global food systems are developing rapidly, and have resulted in a large burden of disease and a high proportion of environmental resource use. We combined global data sources on food supply and trade, environmental footprints, burdens of disease, and vulnerability to climate change to explore patterns from 1990 to 2017. Four distinct patterns of food supply (animal sources and sugar, vegetables and nuts, starchy roots and fruits, and seafood and oils) were matched to health and environmental risks. The animal sources and sugar pattern was found to have the greatest environmental footprint and to be associated with a greater burden of chronic disease than any other pattern, although it was also associated with lower undernutrition. This pattern is globally predominant, but has begun to decrease in higher income countries. Countries where this pattern is predominant are generally among the least susceptible to climate change, whereas more susceptible countries tend to have more sustainable patterns of food supply. More countries that are susceptible to climate change are increasingly exporting a larger proportion than before of their cereals, fruit, and vegetables globally, which will lead to increased risks in global food security. To increase resilience to future shocks, dietary change towards more sustainable patterns should accelerate in high-income countries, and the food systems of the most susceptible countries should be protected.

## Introduction

An insufficient supply of dietary energy and of essential vitamins and minerals including iron, iodine, and vitamin A have historically been the major diet-related causes of ill health. These problems still persist; globally, approximately 800 million people are undernourished in 2017,^1^ and 2 billion people are estimated to have been deficient in essential micronutrients in 2020.^2^ However, substantial advances in crop and livestock production, together with major transitions in the demand for specific foods, have shifted disease profiles around the world. The largest contribution now to the global burden of disease derives from food-related non-communicable diseases associated with overweight and obesity, including cardiovascular disease, diabetes, and cancer,^3^ with 26% of all adult deaths in 2021 related to food. Inequality in the supply of adequate and healthy diets persists and in many countries there is a double burden of undernutrition and non-communicable disease.^4^

The global agricultural sector has, to date, responded successfully to major demographic and socioeconomic transitions by producing more and increasingly diverse foods, but this production has had a notable effect on the environment. Increased requirements for agricultural land, large-scale deforestation, the widespread application of chemical fertilisers and pesticides, unsustainable withdrawal of ground and surface water, air pollution, and degradation of soil quality have led to major changes to ecosystems and biodiversity, as well as making a substantial contribution to global greenhouse gas emissions. Agriculture has been identified as the single greatest contributor to the environmental changes that are breaching planetary boundaries for global environmental sustainability.^5–7^

Warnings over the potential effects of climate change on agriculture started to appear in prominent health journals in the early 1990s.^8^ Crops and animals are sensitive to climate and environmental change, including decreased water availability, increased temperatures, reduced pollination of crops, and increased frequency of extreme weather events. In the absence of adequate food system responses, projected environmental changes are estimated to reduce the global per-person food availability by 3·2% by the mid-century,^9^ with associated reduced nutrient availability and large differences across geographical regions.^10^ Environmental change is a reality in many parts of the world and the evidence that it is having a substantial effect on agricultural systems is growing.^11,12^ The effects are particularly visible in low-income and middle-income countries with food systems that are already susceptible to such change. For example, countries in southern Africa have had some of the largest climate-related harvest failures in the past few decades.^13^ Population growth, dietary transitions, and increasingly lengthy food chains are adding to these growing pressures from environmental change; in particular, the increased use of land that is not suitable for high agricultural productivity and the overexploitation of natural resources, such as water. The combined effect of environmental change and pandemics on the agricultural sector is not known.

For more **information on nutrition-related diseases and death** see https://globalnutritionreport.org/

Increased trade has had economic benefits for nations and food system sectors,^14^ and the expanded diversity of the global food supply has also increased dietary diversity in many national food systems.^15^ However, the effects have been unequally distributed, with many susceptible populations in low-income and middle-income countries not benefitting from international trade policies.^16^ Furthermore, although the increased trade of agricultural products could contribute to enhanced food system resilience,^17^ several unintended consequences have been identified, including the outsourcing of the costs for the environment of agricultural production which have, to date, been poorly managed.^18,19^

In this paper we assembled and analysed global data sources to explore historical and current relationships between food supply and agriculture, the natural environment, and human health, and identify potential challenges for the future provision of sustainable and healthy diets for a rapidly growing global population.

## Methods

### Data sources

We assembled and matched national-level data from 1990 to 2017 from four main publicly available sources for our analysis: first, food supply data from the Food and Agriculture Organization of the UN (FAO); second, data on environmental footprints of different food groups from the Hestia database; third, data on nutrition-related national disease burdens from the Global Burden of Diseases, Injuries, and Risk Factors Study (GBD) study; and fourth, data on vulnerability and resilience to climate change from the Notre Dame Global Adaptation Initiative (NDGAIN).

Information on the food available for human consumption (food supply) at the national level has been systematically collated by FAO (FAOSTAT), with data available from 1961.^20^ These food supply data do not provide an accurate representation of individual dietary intake, because they do not account for within-country inequalities in access to food, household-level food waste, national-level reporting omissions, or the preferences of subpopulations, but they can be used to compare the national food supply over time and between countries, and can act as a proxy for national food system change.^21^ Previous studies have also used these data to show an association of food supply with global disease burdens.^22^ FAOSTAT also provides data on the imports and exports of food groups in tonnes per country per year.

The Hestia database brings together studies that have the estimated environmental footprints of different food products in a single standardised platform, providing both global average footprints and, in some cases, country-specific footprints. The GBD study produces standardised data at the national level on mortality, morbidity, and health risk factors,^23^ which can be matched to individual countries in a given year to estimate their disease burdens from all causes or from specific causes.

For the **Hestia database** see https://www.hestia.earth/

The NDGAIN Country Index summarises the combined assessment of each country’s vulnerability to climate change and readiness to strengthen its resilience, for 181 countries in the world.^24^ The score includes measures of exposure to climate hazards, dependence on climate-sensitive sectors, and adaptive capacity. Applied to the agricultural sector, it reflects both the immediate vulnerability of the sector to the effects of climate change and the probable capacity of countries to adapt effectively.

For the **NDGAIN Country Index** see https://gain.nd.edu/assets/254377/nd_gain_technical_document_2015.pdf

### Analysis

Data from FAO Food Balance Sheets^25^ from 1990 to 2013 representing the availability for human consumption of 18 different food groups (in kg per person per day as a proportion of the total availability of all foods) in 170 countries were the inputs to a principal component analysis that identified the patterns of food supply. Further details on this model have been previously published,^26^ but briefly, the model aimed to reduce the huge complexity of different food groups available in different countries to a few patterns that explained most of the variance in food supply and that could be simply and usefully compared with one another. The resulting food supply patterns do not represent national diets, but rather consistent features of food supply in a given country that might be associated with other variables, such as health and environmental indicators.

We scored countries on their adherence to each food supply pattern in every year from 1990 to 2013 (higher scores indicate a greater adherence to each food supply pattern, with a minimum possible score of 0 and a maximum of 100), and explored changes in scores over time in countries of different income levels. To estimate the association between adherence to different food system types and national environmental footprints, we allocated each country to its predominant food system type in each year from 1990 to 2013 (ie, the predominant food system type in a country could change over time). We used the global average footprint data from the Hestia database for the food groups included from the Food Balance Sheet data to estimate greenhouse gas emissions, freshwater withdrawals, and land use associated with supply of 1 kg of each food group. Individual environmental footprints were transformed to z-scores and averaged to produce a mean standard environmental footprint for each country in each year. We also assessed the association over time of the different food system types with nutrition-related disease burdens using data from a GBD study burdens.^23^

For more **information on different income levels** see datahelpdesk.worldbank.org

To investigate the resilience of national food systems to climate change, we assigned an aggregate score to each country (with 1 representing the most susceptible and 5 the most resilient) on the basis of the continuous scores provided by NDGAIN. Countries were subsequently merged into UN subregions for visualisation purposes. Population statistics from the World Bank (reference year, 2017) were used to calculate the population-weighted average NDGAIN scores by subregion. Global export data were obtained from the FAOSTAT database,^25^ and trends in the exports of major food groups (animal-sourced foods; fruit, vegetables, and legumes; cereals; and starchy roots and tubers) were plotted. The FAOSTAT data were matched with country-specific NDGAIN quintile scores.^24^ Re-exports were removed as their food origins were unreported. Analyses were conducted in Stata version 17.

## Results

**Figure 1:**
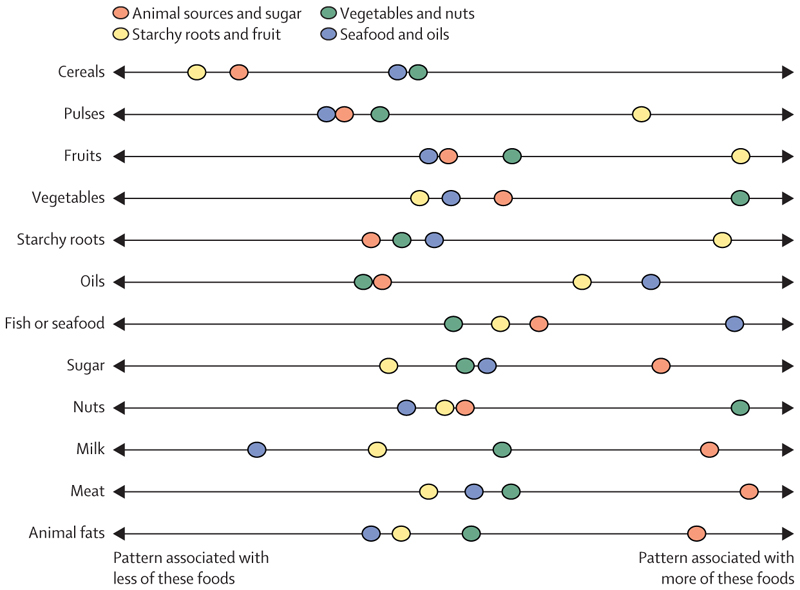
Selected food groups associated with food supply patterns The position of each food supply pattern on the line indicates whether that pattern is associated with a greater or lesser supply of each food group.^25^

Four multi-dimensional food supply patterns (figure 1) explained 89·2% of the total variance in the global food supply.^26^ These patterns were named according to the two food groups most strongly associated with each type: animal sources and sugar; vegetables and nuts; starchy roots and fruit; and seafood and oils.^26^ The vegetables and nuts, and seafood and oils patterns were associated with a greater availability of cereals than all other patterns, whereas the seafood and oils, and starchy roots and fruit patterns were associated with a lower availability of animal-sourced foods (other than seafood).

**Figure 2:**
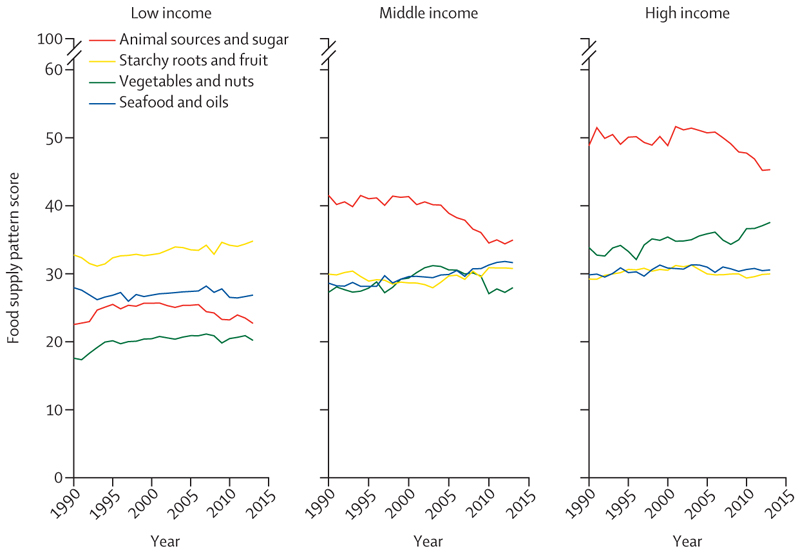
Mean adherence scores of countries (n=170) to four food supply patterns from 1990 to 2013 separated by World Bank income groups Scores were between 0 and 100.

Levels and trends in adherence to the four food supply patterns were quite different across national income groups (figure 2). Most notably, adherence to the starchy roots and fruit supply pattern was highest in low-income countries and showed little evidence of changing over time, whereas adherence to the animal sources and sugar supply pattern was highest in middle-income and high-income countries, although it declined from 2000 onwards. Adherence to the vegetables and nuts pattern showed a steady rise in high-income countries but also an increase in low-income countries, with an unclear trend in middle income countries. The seafood and oils pattern was largely static over time, although there was some evidence of an increase in middle-income countries as the animal sources and sugar pattern declined.

When individual countries were assigned to their predominant food supply pattern in each year and matched to national data on environmental footprints and disease burdens, we found that the animal sources and sugar pattern was predominant in the largest number of countries in 1990 (n=101), followed by the seafood and oils pattern (n=28), the starchy roots and fruit pattern (n=26), and finally the vegetables and nuts pattern (n=15). By 2013 the disparity between patterns had narrowed slightly, although the animal sources and sugar pattern was still the most prevalent (n=96). The starchy roots and fruit (n=27) and seafood and oils (n=26) patterns were still predominant in a similar number of countries compared with 1990, but the vegetables and nuts pattern had increased to be predominant in 21 countries.

**Figure 3:**
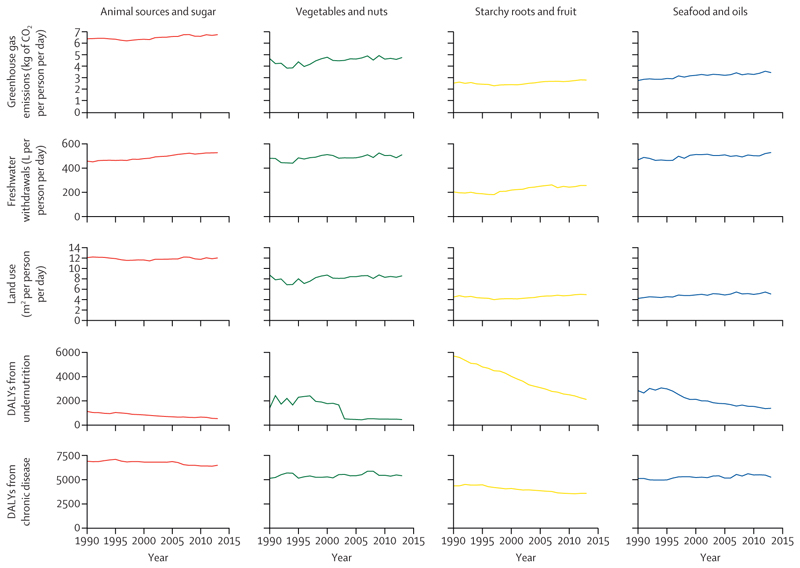
Mean environmental footprints and nutrition-related disease burdens in 170 countries from 1990 to 2013 according to the predominant food supply pattern Plots show change over time in mean national amounts of each indicator, separated according to the predominant food supply pattern in each country. DALYs=disability-adjusted life years.

Having a predominant animal sources and sugar pattern was associated with higher national greenhouse gas emissions, freshwater withdrawals, and land use than any other pattern (figure 3). Countries with a pre-dominant vegetables and nuts supply pattern also had high water use but lower greenhouse gas emissions and land use than the animal sources and sugar pattern, and countries with a predominant seafood and oils pattern tended to have high freshwater withdrawals. In general, the lowest national environmental footprints were found in countries with a predominant starchy roots and fruit pattern, which might partly also reflect lower total food availability in these countries.

Countries with a predominant animal sources and sugar pattern tended to have low disability-adjusted life years from undernutrition and the highest burden from nutrition-related chronic disease (figure 3). In contrast, countries with a predominant starchy roots and fruit pattern had the highest burden of undernutrition (although this rapidly deceased over time), and a low burden of nutrition-related chronic disease. For countries with the vegetables and nuts or seafood and oils pattern as their predominant pattern of food supply, the burden of chronic disease was lower than in countries with a predominant animal sources and sugar pattern, whereas the disease burden from undernutrition showed a marked decline from the mid-1990s onwards. For countries with a predominant vegetables and nuts supply pattern, the disability-adjusted life years associated with undernutrition had reduced to amounts similar to the animal sources and sugar pattern by the early 2000s.

**Figure 4:**
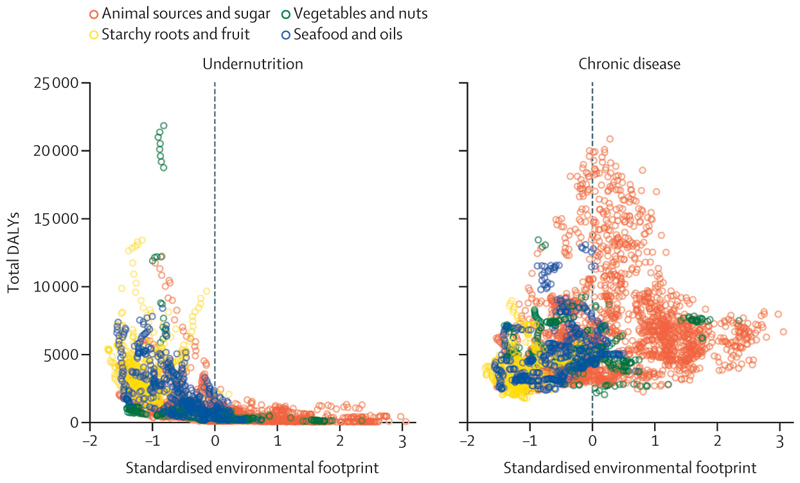
Average national environmental footprints and nutrition-related DALYs by predominant food supply pattern Dots represent individual countries (n=170) in each single year (over 23 years, from 1990 to 2013). Countries in the lower left of the plot have food systems with lower environmental footprints and lower associated nutrition-related DALYs, whereas countries in the top right of the plot have food systems with higher environmental footprints and higher DALYs. DALYs=disability-adjusted life years.

When standard measures of total nutrition-related disability-adjusted life years and environmental foot-prints were plotted against one another for the same countries, the animal sources and sugar food supply pattern had higher environmental footprints than the other patterns, and although this pattern was associated with low undernutrition (figure 4, left panel), it showed a much stronger association with increased nutrition-related chronic disease (figure 4, right panel). In contrast, the food supply patterns associated with the lowest combined environmental footprints and the lowest nutrition-related chronic disease were the starchy roots and fruit and seafood and oils patterns. Countries with a predominant vegetables and nuts pattern showed a wide variability in both health burdens and environmental footprints from their food systems.

**Figure 5:**
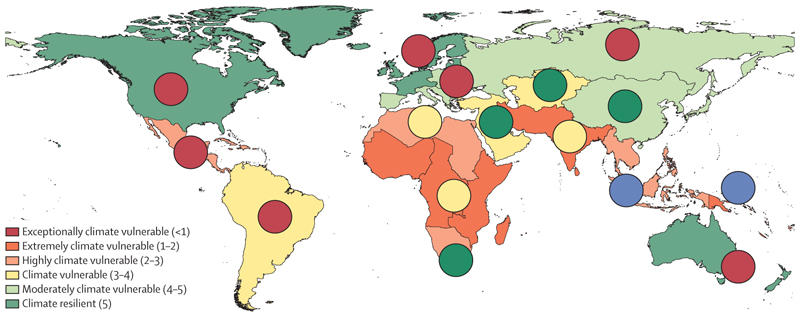
Notre Dame Global Adaptation Initiative Country Index Climate vulnerability in 2017 (population-weighted) by UN subregion, with predominant food supply patterns overlaid Circles represent the predominant food supply pattern in each region in 2013 (red represents animal sources and sugar; green represents vegetables and nuts; yellow represents starchy roots and fruit; and blue represents seafood and oils).

When we scored countries according to their NDGAIN scores for climate vulnerability in 2017 and grouped them by UN subregions,^27^ we found that sub-Saharan Africa, south and southeast Asia, and some small island states (Micronesia and Polynesia) were extremely climate vulnerable (figure 5). The figure shows that countries with the lowest vulnerability to climate change had the food supply patterns with the highest environmental footprints (largely the animal sources and sugar pattern), whereas the countries most vulnerable to climate change generally had food supply patterns with lower environmental footprints (largely the starchy roots and fruit pattern, as well as the seafood and oils pattern). However, there are some examples of countries with lower vulnerability to climate change where more sustainable food supply patterns are prevalent, particularly across western, central, and eastern Asia, where the vegetables and oils pattern is predominant and vulnerability to climate change is moderate.

**Figure 6:**
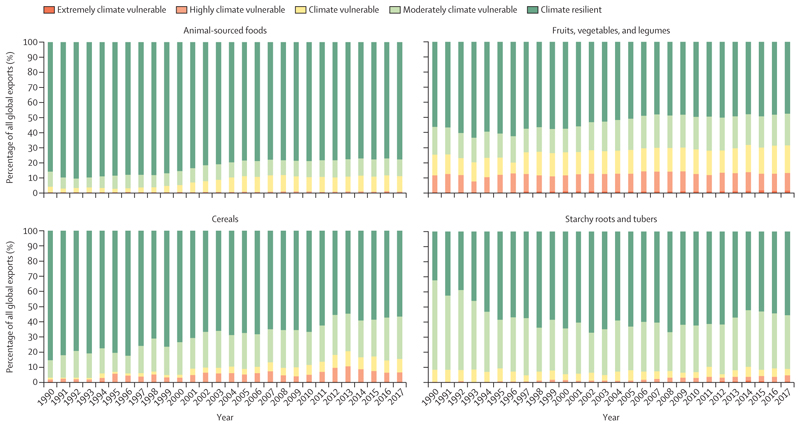
Global exports of four major food groups by NDGAIN climate vulnerability classification of the producing countries (1990–2017)

Global food trade statistics from FAOSTAT when combined with the NDGAIN scores of the countries producing the foods show that the world is increasingly reliant on food production in climate vulnerable countries, with exports from these countries—especially fruits and vegetables and legumes, and cereals—rising as a proportion of the total food trade from approximately 15% to 40% of cereals and from 40% to 50% of fruit and vegetables and legumes from 1990 to 2017 (figure 6). The high dependence of the global supply of fruits and vegetables and legumes on climate vulnerable countries is of particular concern for nutritional security.

## Discussion

This analysis of diverse global data sources has shown that distinct patterns of national food supply are associated with both environmental footprints and health burdens, and that it is generally the countries least vulnerable to the devastating effects of climate change that have the food supplies with the highest environmental footprints. There is also an increasing global reliance on some foods exported by climate-vulnerable countries. However, there are signs of a positive transition towards more sustainable patterns of food supply (particularly the vegetables and nuts pattern) in some high-income countries, along with continuing signs that the burden of undernutrition is being reduced.

The present analysis is limited to an exploration of purely national-level and aggregated trends, and is not able to distinguish subnational relationships or those limited to specific population groups. This analysis also uses food supply data that cannot be considered to represent actual diets, and the noted transitions between food supply patterns do not take account of technological or other innovations that might have occurred in food systems. Correlations between food supply, environmental footprints, and health burdens at the national level are purely ecological and might therefore be subject to confounding factors, although the associations found persisted after adjustment for country income level and total food supply. The NDGAIN index is also limited, because it provides only a national-level overview of vulnerability and does not account for specific areas of a country that might have pockets of vulnerability (and that might also be food-producing areas). However, combining the metrics of the effects on health and the environmental footprints of national food system types in this way can provide new perspectives on the relationship between the environment, food systems, and health.

Over the past 150 years, substantial and widespread transitions in agriculture have brought many benefits to humanity and have been pivotal to the unprecedented improvements in human health and wellbeing. Most notably, except for in conflict zones and humanitarian disasters, the development of agriculture and its ability broadly to keep pace with the demand for dietary energy from a growing global population has meant that widespread and seasonal famines are no longer common.^28^ However, recent events, including the COVID-19 pandemic and global conflicts, have shown that food insecurity can rapidly escalate when trade systems break down. The benefits of the current agricultural systems are in jeopardy if resilience is not increased against these increasing threats, and climate change is likely to provide the next great risk.

A major part of humanity’s current success is built on the unsustainable use of natural resources that, if left unaddressed, will have far-reaching consequences.^29^ The evidence available currently on the health effects and environmental footprints of agricultural transitions and associated dietary changes enhances the ability to reshape, or at the very least reimagine, current agricultural systems to make necessary and sensible adjustments to optimise the benefits of these systems for the environment, food supply, and human health. Major transformations will be required to reduce the negative effects of current food systems in many high-income countries,^30^ which should, as the primary polluters, lead the way. However, there is also the opportunity to identify culturally sensitive and context-specific just transitions^31^ (a type of transition that improves incomes, especially for the poorest, while also being sustainable) for lower income countries. There will be necessary trade-offs, but there is increasing evidence that there are substantial co-benefits from transitions to sustainable and healthy diets,^32^ and further sustainable food supply patterns have been identified here including the vegetables and nuts pattern. The ability of food systems to deliver these sustainable and healthy diets at the global level is in question; and, without dedicated efforts (including tracking emissions based on consumption rather than production and more transparent country-of-origin labelling) to reverse the flow of resources from the most to least vulnerable countries, future food security will be at risk.

In previous decades, evidence on the interlinks between environment, agriculture, food supply, and health has accumulated rapidly and highlighted some areas for immediate action, including reducing consumption of animal-sourced foods in high-income settings. However, to enable evidence-based action, research efforts need to focus on the many evidence gaps. Although analysis at the global level is useful for setting the scene, most decision making is at national and subnational levels. Downscaling methods and metrics to facilitate the analysis of the environmental footprints and health effects of agriculture at national and local scales would enable context-specific and tailored decision making. Attention should be paid to the translational step from research to decision making—for example, by expanding open-access and user-friendly tools, such as the Food Systems Dashboard, to include indicators of system vulnerability to shocks.

For the **Food Systems Dashboard** see https://foodsystemsdashboard.org/

Furthermore, there are many understudied topics in the environment, agriculture, food, and health nexus, in particular the drivers of transitions, and a better understanding of these topics is crucially required to enable the large changes that are needed. The interconnecting concerns associated with agriculture and health cannot be managed without a far stronger evidence base, and the careful assessment and documentation of current effects will be pivotal to future planning and decision making for sustainable and healthy transitions. These processes include the comprehensive mapping and tracking of progress towards initiatives such as the Sustainable Development Goals at the national level to identify unsustainable practices and establish potential leverage points for improvement.^33,34^ Modifying agricultural systems to improve both the environment and health might have unintended negative consequences for some food system actors that should be identified and mapped. Strategies ideally codeveloped with affected stakeholders to reduce, mitigate, and adapt should be closely monitored and evaluations shared to stimulate collective learning.

Finally, sensible agricultural transformations will prove challenging if not backed up with the necessary intersectoral policy framework, bringing together the complex links between food, agriculture, the environment, and health. Historically, agricultural policy has been largely divorced from the considerations of human health, a separation that is most visible in the division of food and health in UN organisations; FAO and WHO. There is increasing awareness in many countries that this approach is no longer serving their populations well. Cross-government initiatives, such as the National Food Strategy in the UK and the plan for food and nutrition security in Brazil,^35^ aim to prioritise healthy, safe, and affordable food, as well as creating systems that can be resilient against future shocks. At a time when agriculture faces enormous pressures in the wake of a global pandemic, there has never been a better opportunity to build new links and redesign agriculture and food systems with a clearer focus on the health of both people and the planet.

For the **National Food Strategy** see https://www.nationalfoodstrategy.org
